# Successful Treatment of Infant Tricuspid Valve Dysplasia With Papillary Muscle-Splitting Technique

**DOI:** 10.1016/j.atssr.2023.03.007

**Published:** 2023-03-31

**Authors:** Yuzo Katayama, Sho Isobe, Tsukasa Ozawa, Takeshiro Fujii

**Affiliations:** 1 Division of Cardiovascular Surgery, Department of Surgery, Toho University Omori Medical Center, Tokyo, Japan

## Abstract

We describe a 3-month-old girl who presented with massive and restrictive tricuspid regurgitation due to shortened chordae attached to an abnormal papillary muscle. The condition was improved by myotomy, which resulted in papillary muscle-splitting technique. We successfully treated tricuspid valve dysplasia in a young infant using a rare procedure based on a thorough understanding of the heart's morphologic features.

Tricuspid valve dysplasia (TVD) is a rare congenital heart condition that usually shows various morphologic features. This makes it difficult to determine the optimal management strategy, particularly for neonates and young infants.

We describe a 3-month-old girl who was diagnosed with severe tricuspid regurgitation (TR) and severe pulmonary valve stenosis after birth. Percutaneous pulmonary valve angioplasty was performed during the neonatal period. Low output syndrome with systemic edema rapidly developed at 2 months, and transthoracic echocardiography revealed an eccentric and massive TR due to the restricted movement of the shortened chordae attached to the septal leaflet (Figure 1A), which necessitated urgent surgery.

**Figure 1:**
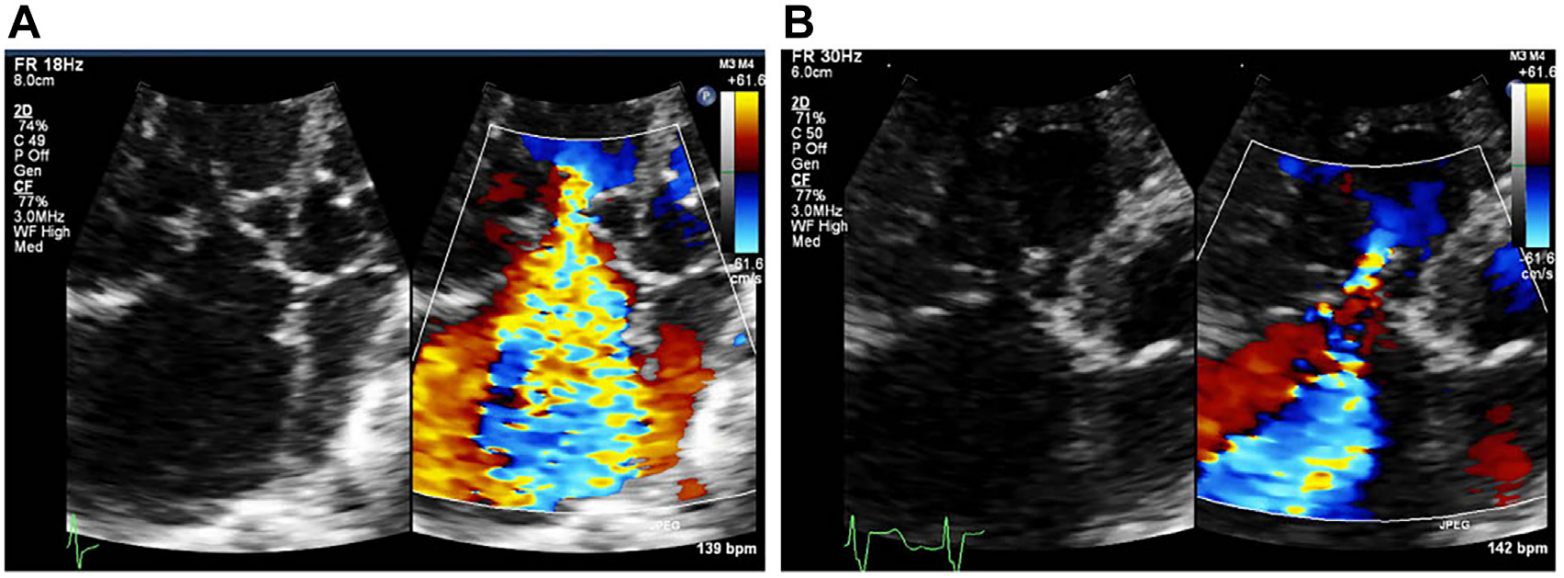
Transthoracic echocardiography revealed tricuspid regurgitation (TR) showing the 4-chamber view before and after surgery. (A) Massive TR due to the restricted movement of the shortened chordae attached to the septal leaflet. (B) Mild TR after the improved coaptation of the septal leaflet.

The operation was performed on full cardiopulmonary bypass. All chordae attached to the septal leaflet were shortened and all attached to the plate-shaped papillary muscle with the leaflet width (Figure 2A). The papillary muscle was not fused to the ventricular septum, and a slight gap was present up to the apex (Figure 2B). The chordae were divided into 3 groups, and the papillary muscles were incised toward the apex between each group (Figure 2C) and outside to optimize the position of the papillary muscles and to extend the chordae (Figure 2D). After confirmation of coaptation improvement and no annular dilation, pulmonary valve commissurotomy was added (Video); intraoperative transesophageal echocardiography showed improvement to mild TR. Transthoracic echocardiography at discharge revealed mild TR and improved coaptation (Figure 1B). She is currently waiting for additional pulmonary valve intervention.

**Figure 2:**
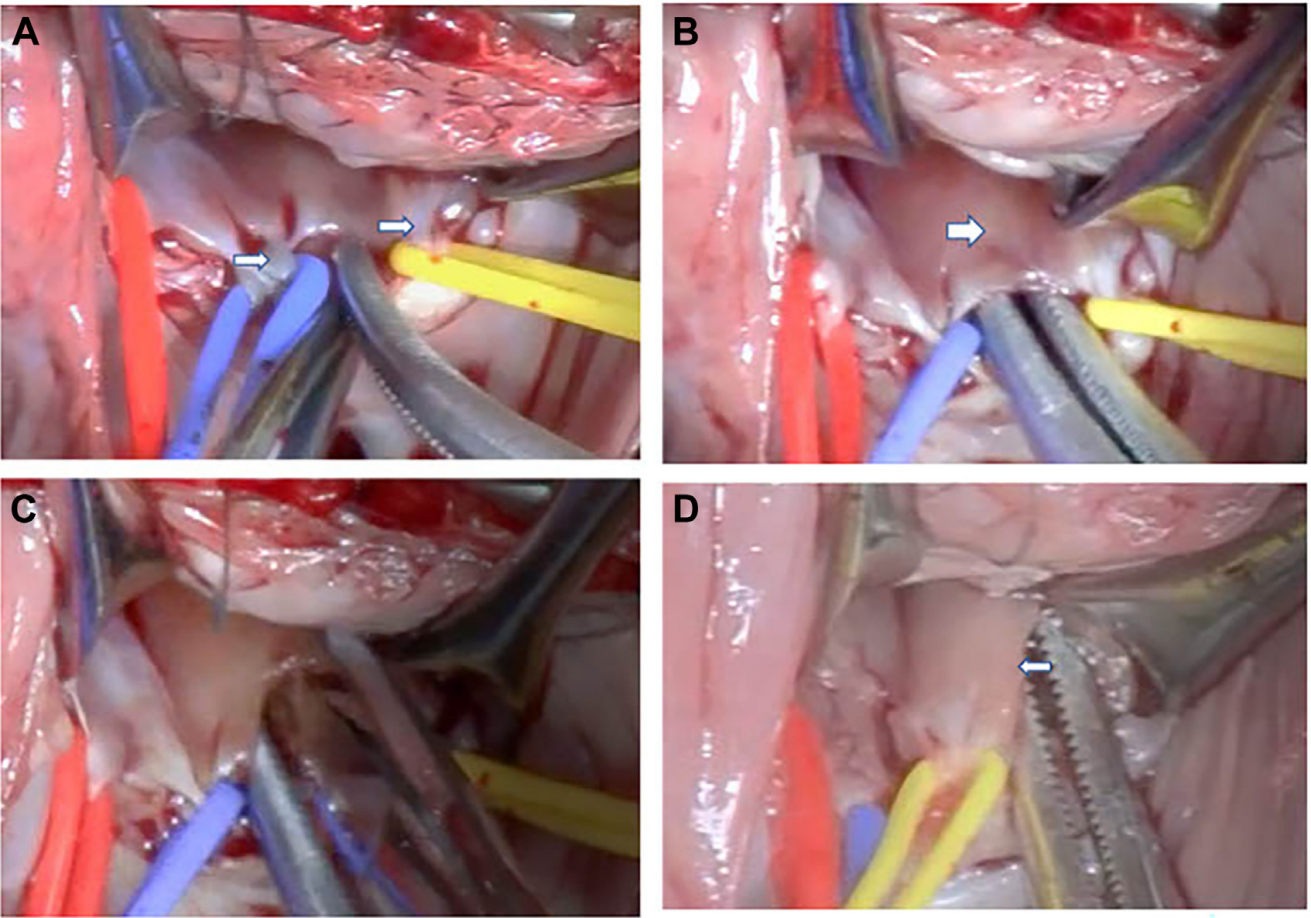
Intraoperative image revealed a morphologic abnormality of the papillary muscle. (A) Shortened chordae (arrows) attached to the septal leaflet. (B) The plate-shaped papillary muscle (arrow) was not fused to the ventricular septum. (C) Myotomy toward the apex between the chordae groups. (D) The position of the papillary muscles was optimized and the chordae were extended (arrow).

## Comment

Tricuspid valve disease in pediatric cases is rare, the most common pathologic process being Ebstein anomaly. In contrast to Ebstein anomaly, TVD has no specific definition, and various morphologic characteristics are described. Possible causes may include primary valve abnormalities or other forms of TVD as in congenitally unguarded tricuspid valve and patients with pulmonary atresia and intact ventricular septum, which can be secondary regurgitation in association with other anomalies as in atrioventricular septal defects, right ventricular outflow tract obstructive lesions, tricuspid valve annular dilation in association with right ventricular volume overload lesions as in congenital coronary arterial fistula with secondary right ventricular enlargement, and Uhl anomaly. Typical features of TVD are annular ring dilation and tethering of the septal leaflet.^1^^,^^2^ The technique for repair of TVD depends on the underlying morphologic appearance, with good results achievable beyond the neonatal period.^3^ With the development of cone reconstruction, the surgical management of Ebstein anomaly has more options, and more stable results have been established.^4^ Cone reconstruction can be applied not only to Ebstein anomaly but also to TVD even in the neonatal period.^5^

Because the infant beyond the neonatal period required urgent surgical intervention for low output syndrome, we initially assumed a combination of simple and common procedures, even with cone reconstruction in mind. The surgical procedure was projected as follows: the shortened chordae are excised; their length is adjusted with artificial chords; if the septal leaflet is insufficient, the autologous pericardium is filled; if annular dilation is observed, Reed technique is added; and if coaptation is still not obtained, edge-to-edge procedure is finally added. Although abnormality of the papillary muscle was not considered, it was assessed intraoperatively that the cause of the concentrated and shortened chordae was morphologic abnormality of the papillary muscle. As the balance between the chordae and the papillary muscles was adequate and the numbers of chordae were sufficient, our strategy was to optimize the position of the papillary muscle laterally and the chordae length longitudinally by extending myotomy to the apex in a well-balanced manner. A 4-lane papillary myotomy was performed up to the same height, and the incision length was determined while confirming adequate coaptation by the backflow test. Although the long-term durability of mitral and tricuspid valve reconstructions with artificial cords in children is good,^6^ it is preferred not to touch the leaflet in young infants.

In our case, low output syndrome was improved by papillary myotomy, which resulted in papillary muscle approximation and chordae elongation. We successfully treated TVD in a young infant using a rare procedure based on a thorough understanding of the heart's morphologic features.
